# Präoperative Nüchternheit bei Kindern – Erfahrungen bei Einführung eines liberalen Nüchternheitsgebotes

**DOI:** 10.1007/s00101-023-01303-2

**Published:** 2023-06-28

**Authors:** Mathias Alexander Gerth, Yannick Maximilian Mußmann, Britta Büchler, Erik Kristoffer Hartmann, Eva Wittenmeier

**Affiliations:** 1 Klinik für Anästhesiologie, Universitätsmedizin, Johannes Gutenberg Universität Mainz, Langenbeckstr. 1, 55131 Mainz, Deutschland; 2 Johannes Gutenberg-Universität Mainz, Mainz, Deutschland; 3 Institut für medizinische Biometrie, Epidemiologie und Informatik (IMBEI), Johannes Gutenberg-Universität Mainz, Mainz, Deutschland; 4 Klinik für Anästhesiologie, St. Marien- und St. Annastiftskrankenhaus Ludwigshafen, Ludwigshafen, Deutschland

**Keywords:** Kinder, Präoperative Nüchternheit, Klare Flüssigkeit, Agitation, Zufriedenheit, Children, Preoperative fasting, Clear fluids, Agitation, Satisfaction

## Abstract

**Hintergrund:**

Das traditionelle präoperative Nüchternheitsgebot für Kinder („6‑4‑2-Regel“) führt häufig zu langen Nüchternzeiten mit unerwünschten Ereignissen (Unwohlsein, Hypoglykämie, metabolische Entgleisungen und Agitation/Delir). An unserer Universitätsklinik wurde daher eine liberale Nüchternheitsregelung mit klarer Flüssigkeit bis zum OP-Abruf („6‑4‑0“) eingeführt.

**Fragestellung:**

Evaluation der realen Nüchternzeiten (Erfolg und Nachhaltigkeit der Umstellung) sowie Einfluss auf verschiedene Parameter wie Zufriedenheit, Agitation, Hypotension und postoperative Übelkeit und Erbrechen (PONV).

**Material und Methode:**

Retrospektive Analyse aus QM-Daten einen Monat vor bis 6 Monate nach Einführung (Juni bis Dezember 2020). Anwendung von deskriptiver Statistik, Odds Ratio und Chi-Quadrat-Test.

**Ergebnisse:**

Es konnten 216 Bögen (44 vorher, 172 danach) ausgewertet werden. Die Nüchternzeit konnte signifikant von 6,1 auf 4,5 h reduziert werden (*p* = 0,034). Bei 47 % der Patienten wurde eine Nüchternzeit ≤ 2h erreicht. Bei zunehmenden Nüchternzeiten im Verlauf war eine erneute Erinnerung nötig. Die Zufriedenheit konnte mit kürzerer Nüchternzeit verbessert werden (Schulnote 2,8 auf 2,2; *p* = 0,004), und präoperative Agitation nahm ab (mod. PAED Skala 1–2 in 34,5 % vs. 50 %, *p* = 0,032). Hypotension trat nichtsignifikant seltener auf (7 % vs. 14 %, *p* = 0,26), während PONV insgesamt zu selten auftrat für statistische Analysen.

**Schlussfolgerung:**

Unsere Auswertung zeigt, dass mit Einführung eines liberalen Nüchternheitsgebots die effektiven Nüchternzeiten reduziert und die kindliche bzw. elterliche Zufriedenheit gesteigert werden können. Ein Monitoring der Nüchternzeiten ist jedoch ratsam, da nach 5 Monaten erneut an die Änderungen erinnert werden musste, um den Erfolg zu bewahren.

**Zusatzmaterial online:**

Die Online-Version dieses Beitrags (10.1007/s00101-023-01303-2) enthält den zugrunde liegenden Erhebungsbogen.

## Abstract (en)

**Background:**

Traditional fasting rules for children prior to elective operations (“6‑4‑2 rule”) often lead to prolonged fasting times with possible adverse events (discomfort, hypoglycemia, metabolic disorders, agitation/delirium). A new liberal fasting policy allowing children to drink clear fluids until being called to the operating room (“6‑4‑0”) was established in our university hospital. This article describes our experiences and retrospectively examines the effects.

**Objective:**

Evaluation of real fasting times before and up to 6 months after the intervention (success and durability of changing fasting policy). Evaluation of impact on outcome parameters, such as patients’ resp. parents’ satisfaction as well as perioperative agitation, arterial hypotension after induction and postoperative nausea and vomiting (PONV).

**Material and methods:**

Retrospective evaluation of methods and interventions from 1 month before to 6 months after changing the fasting policy (June–December 2020). Statistical analysis using descriptive statistics, odds ratio and χ^2^-test.

**Results:**

Of 216 analyzed patients 44 were in the pre-change group and 172 in the post-change group. We could significantly reduce clear fluids fasting times over the 6 months after the intervention (median fasting time: from 6.1 h to 4.5 h; *p* = 0.034) and achieve our aim (fasting time for clear fluids less than or equal to 2h) in 47% of the patients. Fasting times became longer again reaching pre-change intervals in the 4th and 5th month, so reminder measures turned out to be necessary. By reminding the staff we could reduce fasting times again in the 6th month and restore patients’ resp. parents’ satisfaction. Satisfaction was improved by shorter fasting times (median school grade from 2.8 to 2.2; *p* = 0.004; odds ratio for better satisfaction 5.24, 2.1–13.2), and preoperative agitation was reduced (agitation modified PAED scale 1–2 in only 34.5% instead of 50%, *p* = 0.032). In the liberal fasting group, we observed a nonsignificant smaller incidence of hypotension after induction (7% vs. 14%, *p* = 0.26) while PONV was too rare in both groups for statistical purposes.

**Conclusion:**

With multiple interventions we could significantly reduce fasting times for clear fluids and improve patients’ resp. parents’ satisfaction as well as preoperative agitation. These interventions included regular presence in all staff meetings, a handout for both parents and staff, as well as a remark on the anesthesia protocol. Children who were operated on later in the day, benefited most from the new liberal fasting policy as they were allowed to drink until being called to the operating room. Following our experience, we consider simple and safe fasting rules for the whole staff as most important for change management. Nevertheless, we could not reduce the fasting intervals in all cases and had to remind the staff after 5 months to preserve this success. For enduring success, we suggest regular staff updates during the change process instead of one single kick-off information event.

## Hintergrund und Fragestellung

### Historisches und aktuelle Entwicklungen zum Nüchternheitsgebot

Das Nüchternheitsgebot vor elektiven operativen Eingriffen mit Anästhesie („6‑2-Regel“ für Erwachsene, modifiziert als „6‑4‑2-Regel“ für Kinder) schützt vor einer gefürchteten pulmonalen Aspiration und ist eine seit vielen Jahren unveränderte Grundregel in der operativen Medizin [7, 14, 18].

Die negativen Effekte überlanger Nüchternzeiten werden jedoch vernachlässigt; so kommen in der operativen Routine mitunter mehr als 12-stündige Nüchternzeiten für klare Flüssigkeit vor. Alte Dogmen und verbreitete Fehleinschätzungen führen gelegentlich dazu, dass sogar kleine Kinder sehr lange dursten müssen [3, 6].

Mehrere Autoren berichten über die negativen Effekte überlanger Nüchternzeiten, gerade für kleine Kinder, die von Unwohlsein über Hypoglykämien bis zu metabolischen Entgleisungen reichen [3, 6]. Auch eine höhere Delirinzidenz wird diskutiert [13]. Andererseits sind relevante Komplikationen durch Aspiration auch bei verkürzten Nüchternzeiten selten [2, 12], da klare Flüssigkeit nur sehr kurz im Magen verweilt [5] und den pH-Wert nicht verändert [15].

In einem europäischen Consensus Statement wird daher seit 2018 die Verkürzung der Nüchternzeit für klare Flüssigkeit bei Kindern auf 1 h gefordert [17], und 2022 ist eine europäische Leitlinie mit neuen Nüchternheitsregelungen verabschiedet [9] und durch den Wissenschaftlichen Arbeitskreis Kinderanästhesie (WAKKA) der Deutschen Gesellschaft für Anästhesiologie und Intensivmedizin (DGAI) in eine aktuelle S1 Leitlinie umgesetzt worden [19]. Letztere beinhaltet weitere Neuerungen (6‑4‑3‑1-Regel) sowie Vorschläge für eine definierte leichte präoperative Mahlzeit [19].

### Maßnahmen zur Einführung eines liberalen Nüchternheitsregimes an der Universitätsmedizin Mainz

Es konnte gezeigt werden, dass v. a. organisatorische Gründe zu einer Überschreitung der 2‑stündigen Nüchternzeit für klare Flüssigkeit führen [1, 8]. So wurde mangels Rückmeldung über den OP-Verlauf in der Praxis häufig präventiv eine Flüssigkeitskarenz ab 6:00 Uhr eingehalten, auch wenn Operationen erst mittags stattfanden [8]. Um zu verhindern, dass aus Angst vor Verantwortlichkeit der Stationen lieber überlange Nüchternzeiten in Kauf genommen werden, ist hier eine enge Absprache ohne evtl. Schuldzuweisungen notwendig. In Anlehnung an das europäische Consensus Statement entschieden wir uns bereits 2020 vor Erscheinen der deutschen S1 Leitlinie 2022 [19] für eine einstündige Flüssigkeitskarenz bei Kindern. Bei der Umsetzung waren in unserer Klinik die langen Transportwege (Pavillonsystem) mit frühzeitigen Patientenanforderungen und meist zusätzlicher Wartezeit in der OP-Schleuse hilfreich. Allein durch Transport und Wartezeit wurde in der Praxis immer eine leitlinienkonforme Nüchternheit von mindestens 1 h erreicht, sodass wir uns auf das liberale, einfach anzuwendende und jederzeit eindeutige Nüchternheitsgebot „klare Flüssigkeit bis zum Abruf in den OP“ einigen konnten. In Absprache mit den Stationsleitungen definierten wir als klare Flüssigkeit ein Glas folgender, auf allen Stationen verfügbarer Getränke: Wasser, klarer Apfelsaft oder Tee (mit oder ohne Zucker). Für andere Kliniken mit kurzen Transportwegen müssen hier ggf. andere Lösungen (z. B. „Trinken bis auf Widerruf“) gefunden werden, um nicht zu kurze Nüchternzeiten zu forcieren [19].

Dazu wurde eine Informationsbroschüre zur Neuregelung für Ärzte und Pflegekräfte gestaltet, sowie eine vereinfachte Broschüre für die Eltern. Diese wurden im Rahmen der Prämedikationsvisite an die Eltern ausgeteilt; zusätzlich wurde ein Stempel „Neue Nüchternheit Kinder – klare Flüssigkeit bis zum OP-Abruf“ auf dem Narkoseprotokoll angebracht.

Nach Gesprächen mit den Einrichtungsleitern (Pädiatrie und Kinderchirurgie), der Pflegedienstleitung und der Kinderkrankenpflegeschule wurde sowohl in der ärztlichen Mittagsbesprechung als auch bei Stationsleitungssitzungen und Stationsbesprechungen persönlich und mithilfe der Broschüre über die Umstellung aufgeklärt. Das Echo der beteiligten Mitarbeiter, speziell auf den Stationen, war durchweg positiv.

Gleichzeitig wurde die Änderung dafür genutzt, die *postoperative* Nüchternheit zu vereinheitlichen. Nachdem zuvor auf den Stationen unterschiedliche postoperative Nüchternheitskonzepte gelebt wurden, war nun klar formuliert, dass nur zwingende chirurgische Gründe (die auch so eindeutig in der Akte zu dokumentieren waren) eine postoperative Nüchternheit rechtfertigen. So wird jetzt üblicherweise bereits im Aufwachraum Trinken und Essen ermöglicht.

## Studiendesign und Untersuchungsmethoden

Die Einführung wurde vom Qualitätsmanagement (QM) des Zentrums für Kinder- und Jugendmedizin begleitet und retrospektiv ausgewertet. Nachdem bereits einige Arbeiten zu den positiven Effekten liberaler Nüchternheitsregelungen im Kindesalter existieren, wurden von uns folgende Fragen untersucht:

- Wie lang sind die Nüchternzeiten tatsächlich?
- Wie ist der Effekt auf die Zufriedenheit von Patienten bzw. Eltern (Zufriedenheit der Eltern bei Säuglingen; bei Kleinkindern Zufriedenheit der Kinder erfragt über die Eltern oder Zufriedenheit der Eltern; ab dem Schulalter Zufriedenheit der Kinder direkt befragt)?
- Wie ist der Effekt auf klinische Endpunkte wie perioperative Hypotension, Unruhe/Agitation und postoperative Übelkeit/Erbrechen (PONV)?
- Wie lange hält die Information bei den Stationsmitarbeitern, und wie häufig muss evtl. nachgesteuert werden?

Die anonymisierte Fragebogenerhebung durch das QM und unsere retrospektive Auswertung wurden der Ethikkommission Rheinland-Pfalz angezeigt und als nicht weiter beratungsbedürftig genehmigt (2020-15078 vom 17.07.2020).

Es wurden elektive kinderchirurgische Patienten (0 bis 14 Jahre) vom 01.06.2020 bis zum 31.12.2020 untersucht.

Im vom narkoseführenden Anästhesisten auszufüllenden Fragebogen wurden neben Alter, Geschlecht, Gewicht auch die tatsächliche Dauer der Nüchternheit (Zeit ab letzter Flüssigkeitsaufnahme bis Ankunft im OP-Bereich) und die Zufriedenheit erfasst. Ebenso wurde das Auftreten von Agitation/Unruhe präoperativ (Kontakt Anästhesie) und 2‑mal postoperativ (30 min nach Narkoseende und bei Verlegung) mithilfe einer vereinfachten PAED-Skala (s. unten) erhoben, sowie das Auftreten von PONV im Aufwachraum (AWR). Auch die Anzahl der Venenpunktionsversuche sowie eine evtl. Hypotension nach Einleitung wurden erfasst. Unruhe/Agitation und/oder PONV im Aufwachraum wurden von der zuständigen Pflegekraft erhoben.

Der Fragebogen ist im Anhang enthalten.

Für Gruppenvergleiche wurde in der statistischen Auswertung eine „kurze Nüchternheit“ als Nüchternheit ≤2 h definiert (Neuregelung), eine „lange Nüchternheit“ >2 h (alte Regelung). Eine Hypotension lag vor, wenn nach Maßgabe des betreuenden Kinderanästhesisten eine Volumengabe und/oder Katecholamintherapie notwendig war. Um dabei den Einfluss der Sympathikolyse auszuschließen, wurde dies nur ausgewertet, wenn nicht gleichzeitig eine rückenmarknahe Regionalanästhesie angelegt worden war.

Zur Erfassung von Unruhe/Agitation wurde eine vereinfachte PAED-Skala (Pediatric Anesthesia Emergence Delirium Scale – PAED) verwendet (jeweils 0 bis 4 Punkte in den Kategorien „unruhig/ruhelos“ und „untröstlich“). Zur Auswertung wurde jeweils eine Summe der erreichten Skalenwerte gebildet (2-mal jeweils 0–4 bei „unruhig/ruhelos“ bzw. „untröstlich“), somit konnte ein Wert zwischen 0 und 8 generiert werden. Die vollständige PAED Skala [11, 16] besteht aus 5 Kategorien, hier zeigt ein Wert ≥ 12 ein behandlungsbedürftiges Delir an. Unser Ziel bei der Verwendung war die quantitative Erfassung von häufig auftretender Unruhe/Agitation (Items „unruhig/ruhelos“ und „untröstlich“) und nicht eine vollständig durchgeführte Delirdiagnose. Hierzu wären weitere Kriterien wie „Augenkontakt“, „zielgerichtete Bewegungen“ und „Wahrnehmung der Umgebung“ nötig.

Zur Auswertung wurden Methoden der deskriptiven Statistik sowie Odds Ratio und Chi-Quadrat-Test verwendet. Hierzu wurde SPSS (IBM SPSS Statistics for MAC OS, Version 27.0.1.0; IBM Corp., Armonk, NY, USA) benutzt.

## Ergebnisse

Der Zeitraum der Erhebung umfasste einen Monat vor bis 6 Monate ab Einführung der neuen Nüchternheitsregelung. Ausgewertet wurden alle Bogen, bei denen neben Alter, Gewicht, Geschlecht eine Angabe zur Nüchternheit gemacht wurde. Wenn sich bei weiteren Angaben Lücken fanden, wurde hier zusätzlich die Anzahl angegeben. Über einen Zeitraum von 7 Monaten konnten so 216 Bogen bei Kindern von 0 bis 14 Jahren ausgewertet werden, 67,7 % der erfassten Patienten (*n* = 146) waren männlich.

Es wurden überwiegend junge Kinder erfasst, 65,7 % der Patienten (*n* = 142) waren 0 bis 3 Jahre alt. Patientencharakteristika vor und nach dem Wechsel bzw. in Vergleichsgruppen zeigen Tab. 1 und 2.

**Table 1:** 

Erfasste Daten	Vor Änderung	Nach Änderung	Statistik
*–*	*n*	*n*	*–*
Anzahl der Kinder (*n* = 216)	44	172	–
Geschlecht m/w (*n* = 216)	29/15	117/55	–
PONV, AWR (*n* = 216)	0	8	–
Hypotonie nach Einleitung (*n* = 172)	3 (*n* = 35)	15 (*n* = 137)	–
*–*	*Median [25.–75. Perzentil]* *(n)*	*Median [25.–75. Perzentil]* *(n)*	*–*
Alter (Jahre)	2 [1–5,5] (*n* = 44)	2 [1–5] (*n* = 172)	–
Gewicht (kg)	12,5 [9–20,75] (*n* = 44)	13 [10–19,75] (*n* = 172)	–
Nüchternheit (h)	5,5 [3–9,5] (*n* = 44)	3 [1–6] (*n* = 172)	Erster Monat: *p* < 0,001 Gesamter Zeitraum: *p* = 0,021
Zufriedenheit (Schulnoten)	3 [2–4] (*n* = 44)	2 [1–3] (*n* = 161)	*p* = 0,004
Agitation präoperativ (PAED-Skala, Summe)	1 [0–2] (*n* = 43)	0 [0–2] (*n* = 163)	–

Alle Angaben sind als absolute Zahlen (*n*) oder als Median [25.–75. Perzentil] angegeben

*Nüchternheit* Zeit ab der letzten Flüssigkeitsaufnahme bis Ankunft im OP; in unserem Setting erfolgt die Einleitung ca. 30 min nach Ankunft im OP

**Table 2:** 

Erfasste Daten	≤2 h	>2 h	Statistik
*–*	*n*	*n*	*–*
Anzahl der Kinder (*n* = 216)	89^a^	127	–
Geschlecht m/w (*n* = 216)	57/32	89/38	–
PONV, AWR (*n* = 216)	5	3	–
Hypotonie nach Einleitung (*n* = 172)	5 (*n* = 69)	13 (*n* = 103)	*p* = 0,26
*–*	*Median [25.–75. Perzentil]* *(n)*	*Median [25.–75. Perzentil]* *(n)*	*–*
Alter (Jahre)	2 [1–5] (*n* = 89)	2 [1–5] (*n* = 127)	–
Gewicht (kg)	13 [9,75–20] (*n* = 89)	13,5 [10–20] (*n* = 127)	–
Nüchternheit (h)	1 [0,5–2] (*n* = 89)	6 [4–11] (*n* = 127)	–
Zufriedenheit (Schulnoten) (*n* = 205)	2 [1–2] (*n* = 87)	2 [2] (*n* = 118)	OR = 5,24 95 % KI = 2,1–13,2
Agitation präoperativ (mod. PAED-Skala, Summe) (*n* = 206)	0 [0–2] (*n* = 84)	0,5 [0,5–2] (*n* = 122)	*p* = 0,032

Alle Angaben sind als absolute Zahlen (*n*) oder als Median [25.–75. Perzentil] angegeben

*Nüchternheit* Zeit ab der letzten Flüssigkeitsaufnahme bis Ankunft im OP; in unserem Setting erfolgt die Einleitung ca. 30 min nach Ankunft im OP

^a^ 8 Patienten enthalten, die bereits im Monat vor der Umstellung akzidentell eine Nüchternheit ≤ 2 h hatten; diese wurden bei der Gesamtzahl mitgezählt, aber nicht als Ergebnis gewertet

### Nüchternheit davor und danach

Insgesamt konnte nach Umstellung über die Erhebungszeit von 6 Monaten bei 47 % der Patienten (*n* = 81) eine Nüchternzeit ≤ 2 h erreicht werden. In Tab. 2 sind 8 Patienten (^a^) enthalten, die akzidentell bereits im Monat vor der Umstellung ≤ 2 h nüchtern waren; diese wurden bei der Gesamtzahl mitgezählt (daher *n* = 89 in Tabelle), aber nicht als Ergebnis gewertet.

**Figure 1:**
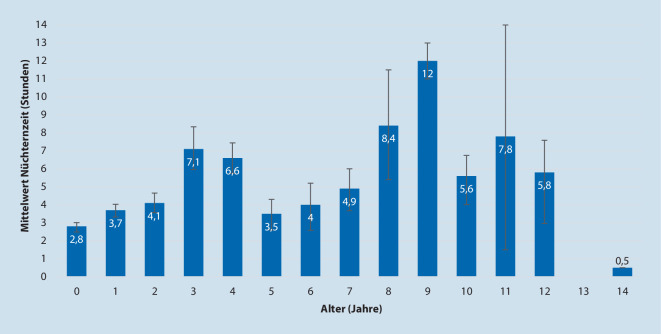

Abb. 1 zeigt die mediane Nüchternheit nach der Einführung, verteilt auf die verschiedenen Altersgruppen:

Wenige Patienten waren sehr lange nüchtern (18 Patienten > 12 h, davon 3 Ausreißer > 16 h). Notfälle und/oder Patienten mit laufender Infusionstherapie waren hier nicht dabei.

**Figure 2:**
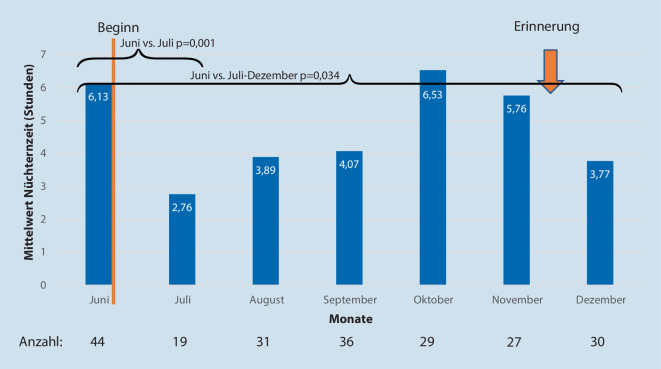

Zu Beginn konnte die deutlichste Verkürzung der Nüchternzeit erzielt werden. So konnte der Mittelwert der Nüchternzeit im ersten Monat der Einführung von 6,1 auf 2,8 h reduziert werden (*p* < 0,001). Trotz nachlassender Compliance im Verlauf konnte auch für den gesamten Beobachtungszeitraum eine signifikante Verringerung der Nüchternzeiten erreicht werden (Mittelwert der Nüchternzeiten von 6,1 auf 4,5 h gesenkt; *p* = 0,021). Den Verlauf der Nüchternzeiten in den jeweiligen Monaten zeigt Abb. 2.

Nach Erkennen wieder zunehmender Nüchternzeiten (diese erreichte im Monat 4 und 5 wieder den Ausgangswert) wurde Anfang des 6. Monats erneut interveniert: Die Intervention umfasste mehrfache telefonische Erinnerungen der kinderchirurgischen Station (pragmatisch bei Abruf der Patienten durch den Kinder-OP-Anästhesisten) sowie eine Erinnerung der Anästhesiesprechstunden an das Anbringen des Nüchternheitsstempels und das Austeilen der Informationsbroschüren an die Eltern. Zusätzlich wurden die beteiligten Stationen persönlich aufgesucht und auch die kinderchirurgischen Kollegen erinnert.

Im letzten Beobachtungsmonat ging so die mittlere Nüchternheit wieder auf 3,8 h zurück (Abb. 2).

Mit kürzeren Nüchternzeiten verbesserte sich die Zufriedenheit (*n* = 205) von Patienten bzw. Eltern erheblich (Monat 1: von Note 2,8 auf 1,9 – Monat 2: 2,2 – Monat 3: 2,5 – Monat 4: 2,8 – Monat 5: 1,7; Monat 6 nach erneuter Intervention wieder 1,8).

Trotz wieder abnehmender Zufriedenheit im Verlauf konnte sie über den gesamten Zeitraum von Note 2,8 auf Note 2,2 signifikant verbessert werden (*p* = 0,004).

Bei einer Nüchternheit ≤ 2 h bewerteten 93,1 % der Eltern ihre Zufriedenheit insgesamt positiv (Schulnoten 1–3) vs. 72 % bei einer Nüchternheit > 2 h. Die Odds Ratio für eine höhere Zufriedenheit (Schulnoten 1–3 vs. Note 4–6) bei kürzerer Nüchternheit betrug 5,24 (95 %-KI: 2,1–13,2).

**Figure 3:**
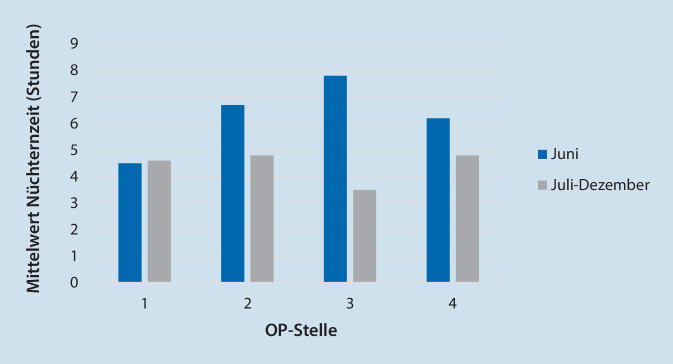

Abb. 3 zeigt die Nüchternzeiten vor und nach der Umstellung, bezogen auf die OP-Stelle (OP-Reihenfolge am Tag).

Es zeigte sich ein Trend zu weniger therapiebedürftiger Hypotension (*n* = 172) nach Narkoseeinleitung bei kürzerer Nüchternzeit (7 % vs. 13 %, *p* = 0,26), hier trat mit einer Odds Ratio von 1,85 keine Hypotonie auf (95 %-KI: 0,63–5,45).

Bei den Agitationswerten (*n* = 206) zeigte der präoperative Wert (erster Kontakt Anästhesie) einen signifikanten Unterschied: Hier waren in der Gruppe mit längerer Nüchternheit 50 % leicht oder stärker agitiert (mod. PAED 1 oder 2), bei kurzer Nüchternheit nur 34,5 % (*p* = 0,032; Odds Ratio 1,90 [95 %-KI: 1,07–3,36]).

PONV (*n* = 216) trat in beiden Gruppen nur selten auf. Von den Kindern mit kurzer Nüchternzeit wiesen 5,9 % ein PONV im AWR auf, bei längerer Nüchternzeit nur 2,4 %. Wegen des insgesamt seltenen Auftretens von PONV und der multifaktoriellen Genese verzichteten wir auf weitere statistische Auswertung.

## Diskussion

Positive Effekte eines liberalen Nüchternheitsregimes sind bekannt [3, 6, 13] und konnten z. T. auch in unserer retrospektiven Untersuchung gezeigt werden, insbesondere die Auswirkung auf die Zufriedenheit und Agitation. Bei anderen Parametern wie Hypotension konnte keine Signifikanz erreicht werden. Die Entwicklung der Nüchternzeiten über den Untersuchungszeitraum zeigt, wie entscheidend wiederholte Erinnerungen an die Umstellung im Rahmen eines „change management“ sind. Alle statistischen Auswertungen sind unter den Einschränkungen einer retrospektiven Untersuchung zu betrachten.

In unserer Untersuchung konnte zu Beginn der Intervention die mittlere Nüchternzeit nahezu halbiert und über den gesamten Erhebungszeitraum bei fast der Hälfte der Patienten in den beabsichtigten Zielbereich (≤ 2 h) gebracht werden. Trotz nachlassender Compliance konnte über den gesamten Zeitraum eine signifikante Verkürzung der Nüchternzeiten erreicht werden.

In unserer Erhebung konnten vorwiegend Daten von jungen Kindern erhoben werden (65,7 %: 0 bis 3 Jahre), der Patientengruppe, die mutmaßlich am meisten von der kurzen Nüchternzeit profitiert [6]. In unserem speziellen Setting (Universitätsklinik im Pavillonsystem mit separater Kinderklinik und verschiedenen operativen Stationen) zeigten sich eindrücklich praktische Probleme: Die Nüchternzeiten konnten zu Beginn der Maßnahme mit intensiver Informationsarbeit beinahe halbiert werden, stiegen aber im Verlauf wieder kontinuierlich an. Durch unser Monitoring der Nüchternzeiten konnten wir entsprechend gegensteuern: Eine erneute Erinnerung im letzten Beobachtungsmonat senkte die mittlere Nüchternzeit hier wieder auf 3,8 h.

Wenige Autoren haben sich bisher mit den praktischen Aspekten der Implementierung beschäftigt: Witt et al. beschreiben Maßnahmen bei erwachsenen Patienten. Sie konnten durch schriftliche Instruktionen an die Patienten und wiederholte Ansprache der Stationen die Nüchternzeit für klare Flüssigkeit von 11 auf 5 h halbieren, blieben jedoch noch erheblich vom Zielwert (2 h) entfernt [20]. Hier findet sich keine Angabe über den zeitlichen Verlauf, d. h., ob ähnlich wie in unserer Untersuchung ein Nachlassen der Compliance zu beobachten war. Isserman et al. beschreiben ein mehr als ein Jahr andauerndes Projekt, bei dem die Nüchternzeiten für klare Flüssigkeit bei Kindern durch mehrfache systematische Interventionen gesenkt wurden [10]. Hier konnte eine mittlere Verkürzung von 9 auf 6 h erreicht werden und eine Nüchternzeit von weniger als 4 h bei > 60 % der Patienten [10]. Hierbei ist hervorzuheben, dass die Umsetzung permanent reevaluiert und dann in insgesamt 4 Zyklen neu justiert wurden. Dabei wurde auch Wert auf positiven Ausdruck gelegt, d. h. zum zeitgerechten Trinken ermuntert, anstatt nur auf die Gefahren bei Nichteinhaltung hinzuweisen [10]. Durch die dauernde Präsenz des Themas und die Fokussierung auf ambulante Patienten in häuslicher Versorgung ist hier auch kein Vergleich möglich, ob und in welchen Abständen das Stationspflegepersonal wie in unserer Untersuchung ggf. nachgeschult werden muss.

Die kürzere Nüchternzeit bewirkte eine deutliche Verbesserung der Zufriedenheit der Patienten bzw. Eltern, die mit zunehmenden Nüchternzeiten im Verlauf der Maßnahme allmählich wieder nachließ.

Al-Robeye et al. konnten in ihrer Erhebung den Zusammenhang von Patientenkomfort und Nüchternzeiten darstellen. So beschreiben sie erhebliches Unwohlsein, Hunger und Durst bei Kindern, wenn die empfohlenen Nüchternzeiten (6‑4‑2-Regel) erheblich überschritten wurden (Mittelwerte 11,7 h für feste Nahrung und 6,9 h für Flüssigkeit) [1]. Engelhardt et al. folgern in ihrer Erhebung bei ambulanten Kindern, dass diese in erheblichem Maße unter viel zu langen Nüchternzeiten leiden und Strategien zur Minimierung dringend notwendig seien [8].

In unserer Untersuchung profitieren v. a. Kinder, die später am Tag operiert werden, von einer kürzeren Nüchternzeit durch die Neuregelung, verglichen mit der alten Regelung (kein Unterschied bei erster OP-Stelle; bei den Stellen 2, 3, 4 unterscheiden sich die Nüchternzeiten vor/nach der Umstellung). Hier zeigte sich der Vorteil der eindeutigen Regelung „klare Flüssigkeit bis zum Abruf in den OP“, wonach auch ohne genaue Kenntnis des OP-Verlaufs noch getrunken werden durfte, anstatt „vorsorglich“ alle Kinder ab 6:00 Uhr dursten zu lassen.

Wir fanden einen Trend zu weniger intraoperativer Hypotension und weniger Agitation im präoperativen Anästhesiekontakt bei kürzeren Nüchternzeiten. Hierbei ist einschränkend anzumerken, dass bei Agitation/Delir neben der Nüchternzeit noch weitere Variablen einfließen (z. B. Ängstlichkeit, Vorerfahrung etc.), die in unserer Untersuchung nicht erhoben wurden.

Eine geringere Rate von Hypotensionen konnten bereits Dennhardt et al. zeigen. Sie empfehlen in ihrer Untersuchung u. a. die Verwendung kohlenhydrathaltiger Getränke, um Hypoglykämien bei Neugeborenen vorzubeugen [6].

Eine Korrelation von langen Nüchternzeiten und postoperativem „emergence delirium“ konnten Khanna et al. zeigen, allerdings unter Sevoflurannarkose und ohne Prämedikation [13]. Eine Untersuchung, die wie in unserer Studie auch präoperative Agitation beschreibt, ist uns nicht bekannt.

In der aktuellen S1-Leitlinie von WAKKA und DGAI sind auch Vorschläge für eine definierte leichte Mahlzeit (> 4 h) enthalten, wie sie in einigen kinderchirurgischen Zentren angewendet wird [19].

Beck et al. konnten in der multizentrischen NiKs-Studie zeigen, dass eine Aspiration von Patientenalter und Notfalleingriff, nicht aber von der Nüchternzeit abhängig war [4]. Die Flüssigkeitskarenz sollte aber bei der Einleitung 1 h nicht unterschreiten, was bei unserem Setting trotz Trinken bis zum Abruf nicht vorkam, da Kinder, die vereinzelt trotz langer Transportzeiten bei Ankunft unter 1 h nüchtern waren, durch eine übliche Wartezeit von ca. 30 min im vorbereitenden OP-Bereich die erforderliche Stunde Flüssigkeitskarenz einhielten.

PONV trat in der liberalen Nüchternheitsgruppe sogar etwas häufiger auf, jedoch insgesamt so selten, dass man, auch bedingt durch das multifaktorielle Entstehen, kaum seriöse Vergleiche anstellen kann.

## Fazit für die Praxis

Neben umfassender Information vor der geplanten Änderung sind besonders folgende Punkte für eine erfolgreiche Umsetzung der durch die neue deutsche S1-Leitlinie empfohlenen einstündigen Flüssigkeitskarenz wichtig:

- Einigung auf bestimmte, auf allen Stationen verfügbare Getränke (z. B. Wasser, Tee oder klarer Apfelsaft) und eindeutige Abgrenzung zwischen klarer Flüssigkeit und Nahrung,
- Gestaltung einer Informationsbroschüre für Eltern und Mitarbeiter, Kennzeichnung des Narkoseprotokolls (z. B. Stempel),
- eindeutige, einfach umsetzbare Regelungen (z. B. „klare Flüssigkeit bis zum Abruf“ oder „klare Flüssigkeit bis auf Widerruf“) für das Stationspflegepersonal treffen, da dieses keine genaue Kenntnis des zeitlichen OP-Ablaufs haben kann,
- eindeutige postoperative Regelung: Kinder sollen i. d. R. postoperativ gleich trinken und essen dürfen,
- Vorhalten von z. B. Getränkepäckchen/Wassereis/Lollis für postoperative Patienten im Aufwachraum,
- regelmäßige Mitarbeiterschulung und enge interdisziplinäre und interprofessionelle Kommunikation,
- eine laufende Evaluation ermöglicht rechtzeitiges Nachschärfen bei erkannten Abweichungen.

## Supplementary Information




