# Supplementary material for: Preoperative fasting in children—Experiences with implementing a liberal fasting policy
Source: Anaesthesiologie. 2023 Jun 28;72(8):565–72. [Article in German] doi: 10.1007/s00101-023-01303-2 (PMC10400684; doi:10.1007/s00101-023-01303-2)
Supplement: Supplementary file 1 [file 101_2023_1303_MOESM1_ESM.pdf]

Erhebungsbogen für den OP „Optimierte präoperative Nüchternheit bei Kindern“

Datum:<br>OP-Nr.:	Alter des Kindes: Jahre; Gewicht: kg<br>Geschlecht: □ m □ w
Nüchternheit präoperativ Nahrung/ Milch:	/ Stunden (vor Beginn Anästhesiemaßn.)
Nüchternheit präoperativ klare Flüssigkeit:	Stunden (vor Beginn Anästhesiemaßn.)
Zufriedenheit Nüchternheitsregelung präop. (Schulnoten 1-6):	□ 1 □ 2 □ 3 □ 4 □ 5 □ 6
Nüchternheit postoperativ:	Stunden (1.Flüssigkeitsaufn. nach ANÄ)

PAED-Skala Agitationskriterien	0 (gar nicht) 1 (ein wenig) 2 (etwas mehr) 3 (viel) 4 (sehr viel)
Unruhe/ Agitation präoperativ (PAED-Skala)<br>Bei Kontakt Anästhesie-Team	ist unruhig/ ruhelos: □ 0 □ 1 □ 2 □ 3 □ 4<br>ist untröstlich: □ 0 □ 1 □ 2 □ 3 □ 4
Unruhe/ Agitation postoperativ (PAED-Skala)<br>30 min nach Narkoseende	ist unruhig/ ruhelos: □ 0 □ 1 □ 2 □ 3 □ 4<br>ist untröstlich: □ 0 □ 1 □ 2 □ 3 □ 4
Unruhe/ Agitation postoperativ (PAED-Skala)<br>Bei Abholung: min nach Narkoseende	ist unruhig/ ruhelos: □ 0 □ 1 □ 2 □ 3 □ 4<br>ist untröstlich: □ 0 □ 1 □ 2 □ 3 □ 4
Agitation/ Delir in Vorgeschichte	□ ja □ nein

PONV im AWR	□ Übelkeit □ Erbrechen
PONV auf Station	□ Übelkeit □ Erbrechen
PONV in Vorgeschichte	□ ja □ nein
Art des Eingriffs	□ abdominell □ HNO □ sonstige
Wartezeit auf Transport (Verzögerung)	Minuten

Anzahl der Versuche i.v. Zugang:	mal<br>□ Maskeneinleitung (schwierige Venensituation)<br>□ bekannter schwieriger Venenstatus
Erste Blutdruckmessung nach Einleitung	/ ( ) mmHg
Therapiebedürftige Hypotension nach Narkoseeinleitung (OHNE Kaudalanästhesie)	□ ja □ nein
Kommentar	

Blutzucker: mg/ dl	Hämoglobin: g/ dl
Standardbikarbonat/ BE: mmol/ l /	pH-Wert:
